# Editorial: Implementation Mapping for selecting, adapting and developing implementation strategies

**DOI:** 10.3389/fpubh.2023.1288726

**Published:** 2023-10-17

**Authors:** Maria E. Fernandez, Byron J. Powell, Gill A. Ten Hoor

**Affiliations:** ^1^Center for Health Promotion and Prevention Research, School of Public Health, University of Texas Health Science Center at Houston, Houston, TX, United States; ^2^UTHealth Institute for Implementation Science, The University of Texas Health Science Center, Houston, TX, United States; ^3^Center for Mental Health Services Research, Brown School, Washington University in St. Louis, St. Louis, MO, United States; ^4^Center for Dissemination and Implementation, Institute for Public Health, Washington University in St. Louis, St. Louis, MO, United States; ^5^Division of Infectious Diseases, John T. Milliken Department of Medicine, School of Medicine, Washington University in St. Louis, St. Louis, MO, United States; ^6^Department of Work and Social Psychology, Faculty of Psychology and Neurosciences, Maastricht University, Maastricht, Netherlands

**Keywords:** implementation science, Implementation Mapping, Intervention Mapping, implementation strategies, adaptation, mechanisms of implementation strategies

The development, or selection and tailoring, of strategies to implement evidence-based interventions (EBIs) is essential for closing the research-to-practice gap and improving health and health equity. Although Intervention Mapping (1) includes planning implementation strategies within its 6-step protocol for planning, implementing, and evaluating multilevel interventions, the standalone process for designing implementation strategies for existing EBIs via Implementation Mapping (IM) (2) was introduced in 2019. It is a helpful tool for guiding the design and tailoring of strategies to enhance intervention adoption, implementation, and sustainment. IM draws from the fields of health promotion and implementation science. It includes five tasks: (1) conduct a needs and assets assessment and identify program adopters and implementers; (2) state adoption and implementation outcomes and performance objectives, identify determinants, and create matrices of change objectives; (3) choose theoretical methods and select or design implementation strategies; (4) produce implementation protocols and materials; and (5) evaluate implementation outcomes. The tasks are iterative, with previous tasks revisited throughout to ensure all implementers, outcomes, determinants, and objectives are addressed.

IM addresses two priorities in implementation science by enhancing the design and/or tailoring of implementation strategies and facilitating a better understanding of the mechanisms through which implementation strategies work (3–5). This Research Topic is dedicated to Implementation Mapping methods, with 15 articles representing a range of settings, topics, and applications (see Table 1).

**Table 1:** Summary of included articles.

**Authors**	**Setting**	**Topic**	**Application of IM**
Savas et al.	Clinical setting: Community Health Worker	Increase breast and cervical cancer screening—SEMM: Salud en Mis Manos	Development of strategies to accelerate and improve implementation fidelity, reach, and maintenance of the SEMM intervention.
Perkison et al.	Clinical setting: Primary care clinics	Adoption of the National Diabetes Prevention Program in primary care clinics	IM was used to systematically identify implementation barriers and facilitators, and design strategies to address those and to develop an adoption, implementation, and maintenance plan.
Valerio-Shewmaker et al.	Clinical setting: community health centers	Blood pressure control; adoption of the Target BPTM program	Identify barriers and facilitators for adoption and implementation of a blood pressure control program and develop strategies to increase program adoption and use.
Domlyn et al.	Urban setting: USA—FQHC	Implementing a computerized strategy of tobacco cessation	Case example for implementation practitioners; feasibility of using IM within an FQHC with limited funds and a 1-year timeline.
Thackeray et al.	Clinical setting: academic health system—physical therapy clinics	Physical activity behaviors among older adults	Development of implementation plan; identifying what physical therapist would need to implement the program, tailored to the needs of the target population.
Watson et al.	Organizational setting	Organizational readiness for implementation of sexual assault prevention	IM used to prioritize readiness goals and develop readiness strategies that will improve implementation of prevention evidence-based interventions for sexual assault prevention.
Markham et al.	School setting in native communities	Adoption and implementation of evidence-based sexual health programs in schools	IM was used to adapt an online decision support system, as well as applying innovative dissemination and implementation strategies.
Jolles et al.	Clinical setting: primary care	Screening for adverse childhood experiences	IM was used to engage diverse partners and guide them through a systematic process that resulted in the development of an implementation strategy.
Lovero et al.	Clinical setting: Primary care clinics of Maputo, Mozambique	Adolescent depression services in primary care	IM was used to design an implementation plan comprising 33 unique strategies targeting determinants at the intervention, patient, provider, policy, and community levels.
Odawara et al.	Organizational setting: small- and medium-sized enterprises in Japan	Prevention of non-communicable diseases	Combined CFIR and IM to develop implementation strategies tailored to the contextual factors identified in the formative study.
Hoskins et al.	Clinical & community setting	HIV medication adherence and care retention	IM used to design a menu of strategies for implementation of an adapted evidence-based intervention for HIV medication adherence and care retention, The process uncovered several challenges. Implementation and effectiveness of strategies developed with IM.
Dickson et al.	Urban setting: USA–FQHC	Improving implementation of two behavioral health programs in a Care Coordination Program	Applied IM for the selection and testing of implementation strategies and integrating additional implementation frameworks within IM.
Davis et al.	National setting: Uganda	Uptake of contact to find and treat individuals with active tuberculosis	Development of a new theory-informed implementation strategy, in combination with the COM-B (Capability-Opportunity-Motivation-Behavior) model and the Behavioral Change Wheel.
Schultes et al.	National setting: Switzerland	Ongoing organized colorectal cancer screening	Evaluation of current state of implementation.
Kang and Foster	Community setting	Community-based rehabilitation by occupational therapists	Applying implementation science in rehabilitation; identification of implementation determinants, mechanisms of action, implementation strategies, and outcome evaluation plans.

Below, we highlight examples of the application of IM (by IM Task) in several of the published studies.

Task 1: Implementation Needs and Assets Assessment: Several articles in this issue describe the use of mixed methods to identify implementation determinants prior to designing strategies to address them. Perkison et al. conducted a needs and assets assessment among frontline staff in community health centers. They employed mixed methods to assess implementation determinants for the National Diabetes Prevention Program (NDPP) by administering a 56-item online survey and conducting 1-h qualitative interviews. The assessments explored determinants at patient, provider, and organizational levels to inform a multilevel and multicomponent implementation strategy to improve adoption and use of NDPP.

Task 2: Adoption and Implementation Outcomes, Performance Objectives, Determinants, and Change Objectives: Thackeray et al. identified adoption and implementation outcomes for use of Coach2Move, a physical therapy intervention for older adults with a musculoskeletal condition. The team focused on adoption and implementation behaviors of clinic managers and physical therapists. They utilized the Consolidated Framework for Implementation Research to examine implementation determinants and described implementation actions (“implementation performance objectives”). They used this information to build a logic model that described the hypothesized mechanisms of action. They also created matrices of change objectives that considered both the specific actions that needed to be carried out to implement the program and determinants that influenced those actions. These matrices helped inform implementation strategy content.

Task 3: Selection of Theoretical Methods and Design of Implementation Strategies: Lovero et al. collaborated with community partners, including policymakers, providers, and representatives from local and non-governmental organizations, to design implementation strategies. They organized collaborative workshops to create implementation research logic models (6) and selected strategies aligned with Expert Recommendations for Implementing Change (ERIC) (7). They also identified new strategies for determinants not well-addressed by ERIC, tailored them to the specific context, and evaluated their priority and feasibility. They specified their strategies using Proctor et al.'s recommendations (8). Two other studies, Savas et al. and Davis et al., exemplified the use of theoretical methods in strategy selection. Savas et al. employed “A Taxonomy of Behavior Change Methods” (9) to guide their approach, while Davis et al. used COM-B and the Behavior Change Wheel (10). Markham et al. demonstrated how to effectively link determinants and change objectives, theoretical change methods (including parameters for their use), and implementation strategies (see Table 4 of that article).

Task 4: Production of Implementation Protocols and Materials: Informed Tasks 2 and 3, Savas et al. provided a design document for their implementation strategy, which provided details to the creative team on the objectives, determinants addressed, theoretical change method, and other guidance needed to develop the material. They also included protocols and final implementation materials.

Task 5: Evaluation of Implementation Outcomes: Kang and Foster used IM to develop implementation strategies for a rehabilitation goal setting and goal management intervention. The IM process informed evaluation plans to explore the impact of implementation strategies using a mixed-methods study. They used self-reported surveys to measure process outcomes, considering the change objectives identified in Task 2. The results of this evaluation can offer valuable insights into the mechanisms of implementation strategies and provide an example of how this information can inform further strategy refinement. An acknowledged limitation was that self-reported outcomes may not always align with objectively assessed performance.

Studies described in the special topics issue focused on various socio-ecological levels and settings including primary health care clinics, Federally Qualified Health Centers (FQHCs), businesses, organizations, schools, a university, and community implementation with community health workers. Two studies describe the application of IM on the national level, in Switzerland and Uganda. See Table 1 for details.

Each article described the IM process, giving varied attention to stating implementation goals, identifying and changing implementation determinants, applying strategies to promote dissemination and implementation, and acknowledging the role of relevant partners. Several studies used IM to integrate the application of several theories and frameworks.

The published articles in this issue show how IM can advance implementation science in several ways including the (1) use of theory in the development of implementation strategies, (2) use of logic models to identify mechanisms, (3) development of implementation research questions, (4) design of studies to evaluate implementation strategies, (5) integration of community engagement in planning strategies to enhance implementation, and sustainment, and (6) planning for broad scale-up and spread.

This Research Topic showcases how IM can contribute to bridging the research-to-practice gap to improve health and health equity. Too many EBIs are not put into practice or are implemented slowly, inequitably, or with poor fidelity. This compromises the potential of research findings in improving healthcare and health promotion efforts. IM outlines a practical method for planning implementation strategies that integrates community engagement, new data, theory and frameworks, and existing evidence. Just as the systematic planning of interventions has improved their effectiveness, IM holds promise for improving the appropriateness, quality, and impact of implementation strategies, which ultimately stands to yield improvements in population health.

## Author contributions

MF: Original revised draft, Review and editing, Writing—original draft. BP: Writing—review and editing. GT: Original draft, Writing—review and editing.
